# The Potential of Photoacoustic Imaging in Radiation Oncology

**DOI:** 10.3389/fonc.2022.803777

**Published:** 2022-03-03

**Authors:** Thierry L. Lefebvre, Emma Brown, Lina Hacker, Thomas Else, Mariam-Eleni Oraiopoulou, Michal R. Tomaszewski, Rajesh Jena, Sarah E. Bohndiek

**Affiliations:** ^1^Department of Physics, University of Cambridge, Cambridge, United Kingdom; ^2^Cancer Research UK Cambridge Institute, University of Cambridge, Cambridge, United Kingdom; ^3^Department of Cancer Physiology, H. Lee Moffitt Cancer Center and Research Institute, Tampa, FL, United States; ^4^Department of Oncology, University of Cambridge, Cambridge, United Kingdom

**Keywords:** photoacoustic (optoacoustic) imaging, radiation oncology, radiotherapy, quantitative imaging biomarker, image guidance, translational research

## Abstract

Radiotherapy is recognized globally as a mainstay of treatment in most solid tumors and is essential in both curative and palliative settings. Ionizing radiation is frequently combined with surgery, either preoperatively or postoperatively, and with systemic chemotherapy. Recent advances in imaging have enabled precise targeting of solid lesions yet substantial intratumoral heterogeneity means that treatment planning and monitoring remains a clinical challenge as therapy response can take weeks to manifest on conventional imaging and early indications of progression can be misleading. Photoacoustic imaging (PAI) is an emerging modality for molecular imaging of cancer, enabling non-invasive assessment of endogenous tissue chromophores with optical contrast at unprecedented spatio-temporal resolution. Preclinical studies in mouse models have shown that PAI could be used to assess response to radiotherapy and chemoradiotherapy based on changes in the tumor vascular architecture and blood oxygen saturation, which are closely linked to tumor hypoxia. Given the strong relationship between hypoxia and radio-resistance, PAI assessment of the tumor microenvironment has the potential to be applied longitudinally during radiotherapy to detect resistance at much earlier time-points than currently achieved by size measurements and tailor treatments based on tumor oxygen availability and vascular heterogeneity. Here, we review the current state-of-the-art in PAI in the context of radiotherapy research. Based on these studies, we identify promising applications of PAI in radiation oncology and discuss the future potential and outstanding challenges in the development of translational PAI biomarkers of early response to radiotherapy.

## Introduction

External X-ray beam radiotherapy (EBRT) is a common and effective treatment for many solid tumors, used as a standalone method or in combination with other treatments, from first line to palliative setting (1). Depending on tumor site and other risk factors, EBRT can be administered as a primary treatment, in the neo-adjuvant setting, to shrink the mass and improve resection success rates, or in adjuvant setting to prevent recurrence (2–4). In conventionally fractionated EBRT, small fractions of radiation dose are delivered to the tumor over several weeks and optimized to spare surrounding healthy tissue (1). Methods that afford higher precision in dose planning and delivery through image-guidance enable higher doses to be delivered in fewer fractions, while achieving similar healthy organ preservation (2, 5).

Response to EBRT is typically assessed by the response evaluation criteria in solid tumors (RECIST) (6) and its derivatives (7, 8) in clinical trials. Such size-based assessments do not account for spatial heterogeneity, can take weeks to manifest, and may be misleading, such as in “pseudoprogression” (9, 10). Similarly, radiation-induced adverse effects in healthy tissue are a key concern for patients undergoing EBRT, but the first clinical signs may take weeks or months to appear (11, 12). In recent decades, the paradigm of response assessment in EBRT has been slowly redefined (10, 13) by the use of molecular imaging in addition to widely used anatomical imaging (14, 15). Molecular imaging can also improve EBRT pipelines by better targeting metabolically active tumor volumes (16–18).

Radiobiological response is strongly influenced by hypoxia, or oxygen starvation, in solid tumors (19, 20). The radiation dose required to achieve a given biological effect is up to 3-fold higher in hypoxic than in normoxic conditions (21). Conventionally fractionated radiotherapy can partially mitigate this through inter-fraction reoxygenation. In hypofractionated courses, such as stereotactic body radiotherapy (SBRT) and stereotactic ablative radiotherapy (SABR), radioresistance associated with hypoxia has been shown to increase in preclinical and modeling studies (22–24), potentially as a result of the partial loss of reoxygenation and the induced vascular damage, subsequently leading to oxygen deprivation. Radiotherapy regimens delivered in shorter timeframes make the need for adequate tumor oxygenation even greater. Moreover, tumors often display substantial spatial and temporal heterogeneity in hypoxia (20, 25), yet our ability to account for this phenomenon in treatment planning and response monitoring is fundamentally limited.

Current molecular imaging modalities afford some insight into the spatial distribution of tumor hypoxia (26). For example, tracers such as fluoromisonidazole (^18^FMISO) in positron emission tomography (PET) can map tumor hypoxia and adjust dose escalation (16, 17) and de-escalation (18) accordingly. PET-CT scans are not typically performed at multiple timepoints, however, because of isotope cost, scanning time, and additional radiation exposure that needs to be justified. Magnetic resonance imaging (MRI) has also shown potential as a non-ionizing modality for defining sub-volumes for escalation of radiation dose based on diffusion (27, 28) and perfusion (29–31) biomarkers, or for predicting response with oxygen-sensitive MRI techniques using tissue and blood oxygen level dependent (TOLD/BOLD) signals with oxygen (32) or carbogen gas breathing challenge (33), reviewed elsewhere (34). Nonetheless, these methods have limited spatio-temporal resolution, comparatively long acquisition times, and may require exogenous contrast agents, with associated toxicity (35, 36). Furthermore, they have limited capability for deployment in conventional linear accelerator rooms, except through combined MR—linear accelerator systems (37, 38) or novel PET—linear accelerator systems (39, 40), which are currently limited by high cost and complexity (41).

Photoacoustic imaging (PAI) is a clinically emerging localized imaging modality that enables affordable, real-time interrogation of oxyhemoglobin (HbO_2_) and deoxyhemoglobin (Hb) in tumors at high spatio-temporal resolution (42, 43). Based on the absorption of non-ionizing optical radiation (44–48), and associated generation of acoustic waves, PAI systems are readily combined with ultrasound given their shared signal detection schemes and provide intrinsically multi-modal imaging to up to approximately 5 cm depth with current technologies (49). When using multiple wavelengths for imaging and applying spectral unmixing algorithms, PAI data can be used to resolve endogenous imaging biomarkers related to total hemoglobin (THb = Hb + HbO_2_) and blood oxygen saturation (sO_2_ = HbO_2_/THb). sO_2_ measured with PAI has been shown to correlate with tumor hypoxia, using *ex vivo* histology as the reference standard (44, 45, 50), demonstrating the potential of PAI to provide surrogate non-invasive biomarkers of tissue hypoxia. With the introduction of exogenous contrast agents, it is also possible to directly report on tumor tissue pO_2_ (51). Moreover, PAI is scalable for non-invasive assessment of single capillaries and even red blood cells (52) and can also be used to extract information on blood flow (53). PAI has thus been proposed for application in superficial tumors to improve: radiation dose delivery and scheduling; patient stratification; and therapy response and radiation side effects monitoring. Here, we summarize the potential of PAI as a fast, portable and affordable tool for monitoring of key radiobiological processes across different length scales, with a particular focus on vascular changes in normal and tumor tissue in response to radiation.

## Potential Uses of Photoacoustic Imaging in Radiotherapy

### Measuring and Monitoring Tumor Response to Radiotherapy With Photoacoustic Imaging

Doses of ionizing radiation delivered in clinical EBRT can induce acute endothelial cell dysfunction, blood vessel disruption, and mitotic catastrophe resulting in apoptosis, which can lead to secondary tissue necrosis (54). When entering tissue, low-linear energy transfer (LET) ionizing radiation, such as X-rays used clinically at MeV energy levels, produces free radicals through the radiolysis of water. If oxygen is present, free radicals form highly reactive peroxyl radicals, which lead to DNA damage and subsequently, cell death (21). In the absence of oxygen, free radicals can be neutralized by interacting with hydrogen or by electron donation, consequently minimizing radiation damage (55). The permeability of capillaries is enhanced after dose delivery and platelet aggregation and microthrombus formation is induced. Altered perfusion can often result, which may in turn cause hypoxia and tumor necrosis, affecting the tumor cell kill of further radiation fractions (56). The timescales and the extent of the change in blood flow to the tumor and in tissue reoxygenation are dependent on many factors, including dose fractionation and have yet to be systematically studied. At SBRT and SABR regimes (>10 Gy/fraction), perfusion changes have been observed in some preclinical studies (57). For instance, in DCE-MRI derived perfusion measurements decreased 2 h post-20 Gy delivery in orthotopic brain tumors in rats (58) although they did not change significantly in subcutaneous lung tumors post-12 Gy (59).

**Figure 1:**
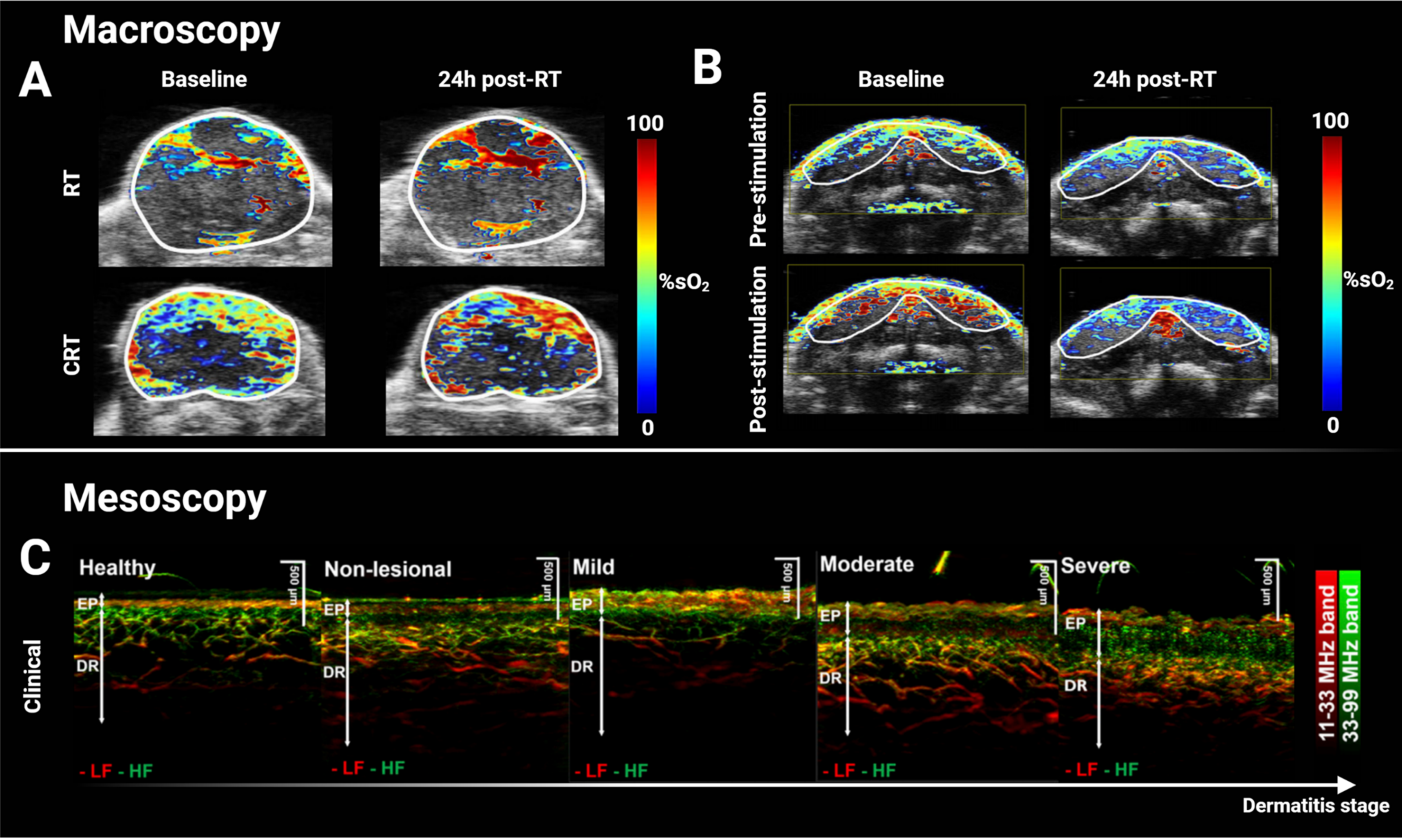
Macroscopic and mesoscopic photoacoustic imaging can monitor treatment-induced vascular changes and disease stage. **(A)** Multispectral optoacoustic tomography (MSOT)-derived quantitative blood oxygen saturation map (sO_2_) overlaid on co-registered ultrasound axial slice of a head & neck patient-derived xenograft tumor before and after a single dose of 15 Gy (top panel), and before and after combined 7.5 Gy of radiotherapy and administration of chemotherapeutic cetuximab. Increased sO_2_ 24h after treatment was associated with decreased tumor volume two weeks later. **(B)** Hemodynamic stimulation challenge of salivary glands before and after a single dose of 15 Gy with decreased change in sO_2_ response post-radiotherapy suggesting radiation-induced damage. **(C)** Clinical XZ maximal intensity projection of mesoscopic PAI of graded atopic dermatitis in human skin. Vascular and structural scoring could accurately grade dermatitis and such score could potentially be translated for grading radiation-induced toxicity in RT. Panels **(A, B)** adapted from Rich et al. (60), and panel **(C)** adapted from Yew et al. (61). EP, Epidermis; DR, Dermis; LF, low frequency; HF, high frequency; RT, radiotherapy; %sO_2_, percent blood oxygen saturation; CRT, chemoradiotherapy.

PAI has been examined in the context of EBRT response assessment in preclinical cancer models. Tumor sO_2_ was demonstrated to be an early biomarker of EBRT response in patient-derived xenografts of H&N cancer, with higher sO_2_ being predictive of response to single dose radiation delivery (**Figure 1A**) (60, 62, 63). Interestingly, increased THb levels during fractionated EBRT were associated with better treatment outcomes (64) and tumors responding to radiation had decreased THb in the early days post-EBRT (64, 65), suggesting PAI can evaluate both tumor sensitivity and early treatment response. Importantly, a dose per fraction of 3 Gy/day was sufficient to cause a significant sO_2_ change as early as 3 days into the treatment course (64), rather than waiting weeks for changes in tumor size to manifest.

EBRT also has the potential to stimulate tumoral neovascularization, as a result of the radiation-induced acute inflammatory response shortly after dose delivery (66). Therefore, blood flow to the tumor and tissue oxygenation may be transiently increased in the few hours following a dose of EBRT, potentially leading to an increase in sO_2_ and THb. After EBRT, Hysi et al. reported increased sO_2_ both 2 and 24 h after a single 8 Gy dose of radiation (67), in an SBRT-like regimen. Interestingly, this also correlated with an increase in the expression of the endothelial marker CD31^+^ area measured *ex vivo*, compared to non-treated controls (67). Days after the end of EBRT, sO_2_ and THb were found to be decreased compared to pre-treatment scans (64, 65). PAI has previously shown that a similar pattern of changes following antiangiogenic treatment may be attributed to vascular normalization, suggesting this mechanism could be observed also in the context of ablative EBRT (68). Since reoxygenation may be crucial for the treatment effectiveness of SBRT fractions (23), PAI offers an opportunity to noninvasively and longitudinally evaluate the timing at which tumors reoxygenate to plan fraction deliveries then.

Dynamic contrast-enhanced (DCE)-PAI has also been assessed as a marker of radiation response (63) using exogenous indocyanine green (ICG) to detect perfusion (44, 69). DCE-PAI was achieved with perfusion quantification based on a two-compartment Tofts model (70) analysis of ICG uptake, finding a promising relationship between decreased perfusion 24 h after a single dose of 10 Gy and early treatment response in xenograft models (63). Similar trends were reported when changing the breathing gas of the mouse from air to 100% oxygen while imaging and measuring the change in sO_2_ (ΔsO_2_) (44, 45), which decreased after treatment (63). PAI using ICG is highly applicable in a clinical setting, since ICG is a clinically approved agent and DCE-PAI has already been shown to be feasible in humans to image finger vasculature (71), to image lymphatic vessels of the lower (72) and upper limbs (73) in 3D, and used to assess metastatic status of lymph nodes in melanoma with PAI in humans (74, 75).

**Figure 2:**
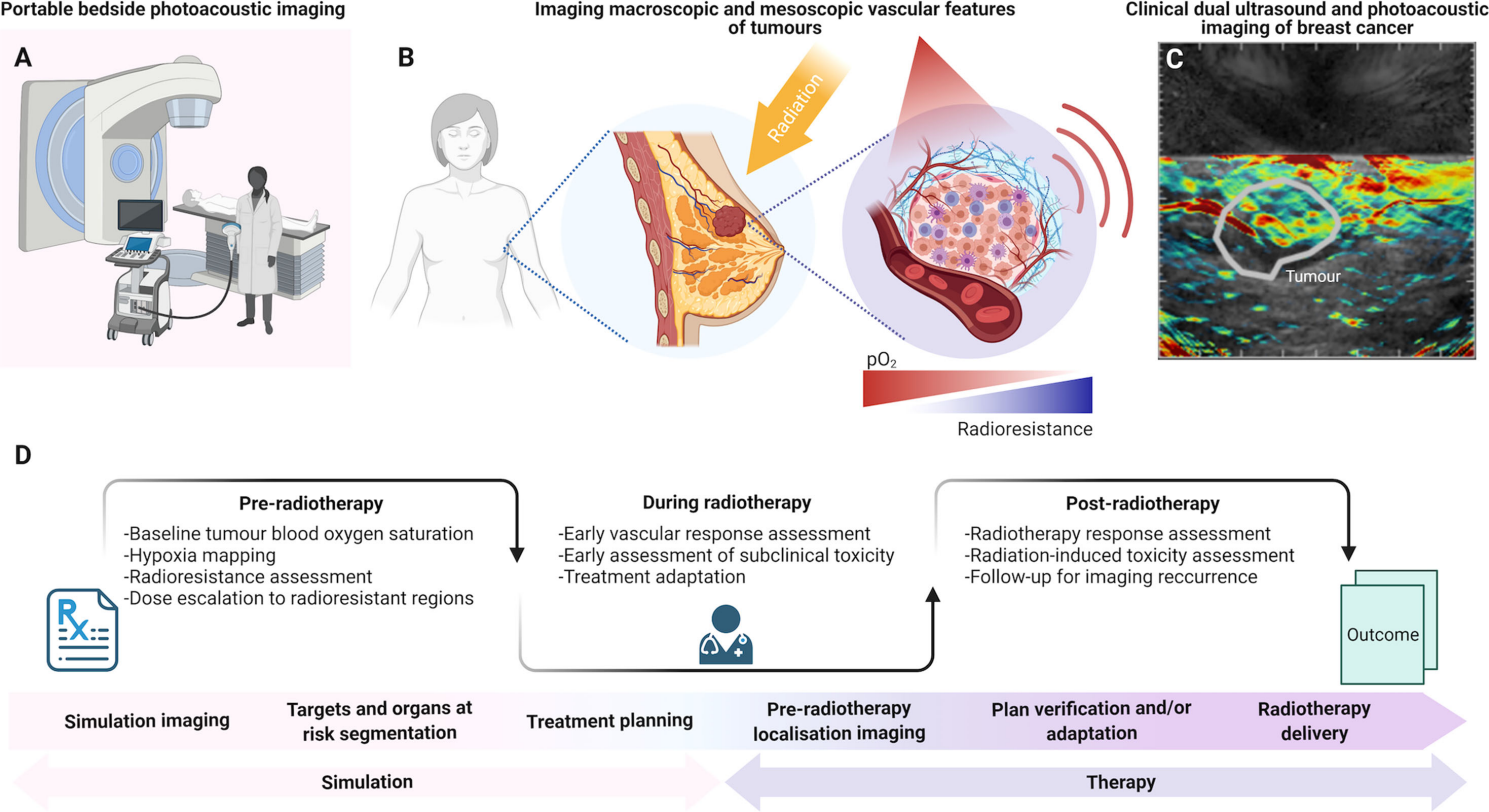
The potential role of photoacoustic imaging in the clinical radiotherapy framework. **(A)** Portable bedside PAI can be employed in-room before and/or after RT fractions due to its accessibility, portability and fast acquisitions. **(B)** PAI could map vascular features of tumors across scales, including blood oxygen saturation. **(C)** Dual ultrasound and PAI systems provide combined anatomical and molecular imaging features. **(D)** PAI could be introduced in the clinical RT framework pre-treatment, for diagnostics and pre-operative patient stratification, or for predictive imaging in parallel with CT simulation for radiation dose modulation. During radiotherapy, PAI could be used for monitoring response in the treatment room. After radiotherapy, PAI could further monitor tumor response based on blood oxygen saturation evaluations, which have been associated with local tumor control. PAI could also provide information for response assessment and insights into radiation-induced toxicity at early timepoints. Panel **(C)** provided in kind by Dr. Oshaani Abeyakoon. Created with BioRender.

In the current workflow for image-guided EBRT, cone-beam CT, anatomical MRI and ultrasound are all used to ensure correct localization of the tumor target and critical organs at risk structures. Additional imaging techniques that provide physiological and biological information regarding tumor and normal tissue response, namely, PET and MRI, are more often combined with standard image guidance in the context of research studies. Considering translation to a clinical context, PAI systems can be fast, cheap and portable, which could enable in-room imaging at the bedside compared to these other functional or molecular imaging modalities (**Figure 2A**). By scaling the resolution linearly with penetration depth (76), PAI offers a flexible approach to imaging vascular features *in vivo* (**Figure 2B**). PAI systems are capable of capturing information on Hb and HbO_2_ content at sub-100 µm resolution in individual blood vessels at superficial (~1 mm) depths (77), or in whole tumors at few cm depths (45, 50). PAI is also readily combined with ultrasound (**Figure 2C**), which provides intrinsically co-registered anatomical information. The spatial resolution scale achievable with PAI provides a distinct understanding of vascular features in the tumor microenvironment locally, compared to PET for instance. While the length scale of quantitative PET has shown potential for voxel-level dose painting in precision radiotherapy, the finer length scale of PAI both at the macroscopic and mesoscopic scales could potentially provide a mechanistic understanding of tumor vasculature response to EBRT. Moreover, PAI measurements at multiple timepoints are much more feasible during a course of fractionated EBRT than PET or MRI, as PAI can be performed using a portable device in the radiotherapy department and even on-set in the treatment position. PAI systems could be readily deployed between fractions to detect changes in sO_2_ that could indicate response, thus providing initial radiation response assessment in-room during radiation fraction delivery. PAI measurements taken in real-time at bedside could inform on oxygen depletion through induction of DNA double strand breaks and the presence of hypoxia, indicating a need for dose modification (63). Although traditional fractionation of ~2 Gy daily, routinely used for these tumors, does not lead to the dramatic vascular response caused by ablative regimes (55), the overall length of the treatment is expected to generate measurable changes, and also providing an opportunity for mid-course adaptation. Nevertheless, further preclinical studies are needed to thoroughly examine modulation of PAI biomarkers in response to both conventional and hypofractionated regimes, in order to better estimate its clinical applications and potentials in the different steps of the radiotherapy framework (**Figure 2D**).

### Targeting Intratumoral Hypoxia With Photoacoustic Imaging Guidance

Intratumoral heterogeneity complicates treatments in radiation oncology. The presence of focal hypoxia is clinically associated with cellular heterogeneity, genomic instability (78, 79), poor prognosis (80) and importantly, poor response to EBRT, particularly in cancers of hormone-sensitive tissues such as the breast (81, 82). Cells within these regions of focal hypoxia will activate hypoxia-inducible factors, which drive the transcription of multiple genes involved in cell growth, metabolism and angiogenesis (48, 83). One such factor is the vascular endothelial growth factor (VEGF), which plays a central role in stimulating endothelial cells to proliferate, sprout and form new blood vessels (84). Overexpression of VEGF often leads to an imbalance of pro- and anti-angiogenic factors, which results in a chaotic and heterogeneous network of blood vessels, namely, many immature vessels with poor pericyte coverage, irregular branching and a tortuous morphology (85, 86). Tumor vascular networks are consequently often poorly perfused, which can generate additional acute and transient hypoxia dynamics further associated with radioresistance (87).

Local tumor control with EBRT can be improved in patients with well-oxygenated tumors (88). The ability to differentiate normoxic from hypoxic tumor regions could thus improve prediction of EBRT outcomes (89), while appropriate image-guided radiation dose modulation could enhance EBRT cancer cell kill by enabling dose escalation to hypoxic regions (90). Unfortunately, while tumor hypoxia has been known as a key limiting factor in the efficacy of radiation for decades, it has yet to be incorporated in the clinical pipeline (91) due to challenges with availability and complexity of the current clinical approaches to hypoxia imaging.

Imaging the aberrant tumor vasculature with PAI has been proposed as a surrogate means to interrogate tumor hypoxia. Preclinical studies in mouse models of cancer have demonstrated that tomographic PAI measurements of THb and sO_2_ can inform on the heterogeneity of tumor hypoxia at ~200 µm resolution (45, 47, 48, 50, 92–97). PAI reveals lower sO_2_ in tumors compared to normal tissue, which is due to the imbalance of blood oxygen supply and tissue oxygen consumption in tumors (94). In multiple cancer mouse models, PAI estimation of sO_2_ correlated negatively with tumor hypoxia, validated *ex vivo* (50, 94–96). The PAI biomarker ΔsO_2_ assessed under gas challenge enables a further robust assessment of the complex dynamics of tumor vessel perfusion, permeability and vasoactivity (44, 45). Importantly, low sO_2_ and ΔsO_2_ spatially correlate with regions of tissue hypoxia and necrosis (44, 95). Taken together, these studies suggest that PAI maps may reveal intratumoral hypoxia at sufficient resolution to guide dose escalation or de-escalation assessments in a targeted therapy approach.

Key targets for potential deployment of PAI in the context of EBRT would be in head and neck (H&N) and breast cancers. Ultrasound is already recommended in H&N cancer to detect and delineate thyroid masses or tumors arising in the neck, and to identify local adenopathy in lymph nodes of the neck (98) before EBRT may be prescribed. In recent meta-analyses, ultrasound has also been recommended in breast cancer for palpable mass detection, especially in low-resource settings (99), and also in addition to mammography, providing increased sensitivity (100). Moreover, clinical PAI mammoscopy has been investigated in a diagnostic context (101) to: enable patient stratification based on intratumoral vascular features characterization (102–104); distinguish molecular subtypes (105); and for diagnostics in patients with dense breasts (103, 106), providing improved lesion detection when combined with integrated ultrasound system (107). Dual ultrasound and PAI systems have also demonstrated higher THb in patient breast tumors compared to normal tissue (108) and enabled visualization of vessels radiating from the tumor mass (109).

Since both H&N and breast cancers are widely treated with EBRT, PAI-based assessment of radiation response is already being investigated in registered and recruiting clinical trials on H&N cancer (ClinicalTrials.gov ID NCT04428515, NCT04110249, and NCT04437030). Nonetheless, the introduction of PAI to escalate dose to hypoxic regions in EBRT requires image co-registration between handheld PAI and planning CT, and would also benefit from co-registration of PAI between fractions to observe local differences in oxygenation. Ultrasound has been investigated for inter-fraction motion management in radiotherapy (110, 111) and fusion to CT for different applications (112, 113). Some promising studies have identified ways of matching skin surface and organ edges on both ultrasound and CT contrasts to co-register images with good similarity in the context of radiotherapy simulation (114, 115) and with probe tracking for intrafraction guidance (116, 117), with an extensive review reported elsewhere (118). Accurate mapping of regions on PAI to planning CT requires the registration of PAI/ultrasound system to the room coordinates using probe localization in the CT simulation suite or in the treatment room (116, 118, 119). Nevertheless, promising findings in the context of ultrasound suggest that this challenge is not insurmountable. Overall, intra- and interfraction PAI monitoring of the tumor microenvironment opens new avenues for live assessment of tumor response to EBRT and dose adjustment based on local differences in intratumoral oxygenation, potentially increasing treatment control, especially in hypoxic tumors.

### Radiation-Induced Toxicity Assessment With Multi-Scale Photoacoustic Imaging

Healthy skin toxicity is a common side effect of radiation, namely, acute dermatitis, burns and inflammation (120), and also chronic changes that may be permanent. Their rapid diagnosis and characterization are crucial for effective control of adverse radiation effects. Unfortunately, the first clinical signs may take weeks or months to appear (11). As skin damage appears during the course of fractionated EBRT, some early signs in the skin vasculature could be detected prior to the appearance of erythema (~2 weeks) as damaged cells migrate to skin surface, or dry desquamation (i.e., skin peeling, ~4 weeks).

At present, clinical management of radiation-induced skin side effects with the Radiation Therapy Oncology Group (RTOG) scoring criteria is limited to the subjective visual assessment of visible clinical signs over weeks (11). For early detection of adverse effects of radiation to healthy skin, changes in HbO_2_ distribution have been shown to precede clinical symptoms, detectable with cutaneous blood flow measurements (121) and characterized *in vivo* with optical imaging modalities, namely, two-photon microscopy (122), diffuse optical tomography (123), and diffuse reflectance spectroscopy (124, 125). Similarly, B-mode ultrasound has already been studied in the context of radiation-induced toxicity, showing predictive parameters consistent with RTOG scores clinically (126).

The addition of PAI contrast to ultrasound parameters for direct measurements of vascularization and blood oxygenation using dual PAI/ultrasound systems or superficial PAI alone, may further assist evaluation of the early signs of acute radiation-induced toxicity and allow for their effective treatment. For example, in a salivary gland stimulation challenge conducted in mouse models, a decrease in sO_2_ change between measurements taken before and after salivary stimulation post-EBRT was associated with radiation-induced salivary gland toxicity in a murine model assessed with macroscopic PAI (**Figure 1B**) (60). Mesoscopic implementations of PAI can achieve ~20 μm in-plane resolution up to ~3 mm in depth for skin imaging (52, 127), indicating potential for clinical skin toxicity assessment (128). Preliminary work on skin atopic dermatitis grading (61) showed that combining PAI mesoscopy-derived total blood volume, average vessel diameter, and ratio of low to high frequency signals gave a discriminating signature for atopic skin dermatitis grade (**Figure 1C**) (129). Beyond vascular imaging, the emerging capabilities of PAI for fibrosis imaging (129–131) may aid characterization of this late-stage skin toxicity manifestation (12, 120). In addition, PAI has shown promise for assessment of burns (132), wound healing (133, 134), and skin disorders such as psoriasis (135), all of which present with features similar to those in radiation-induced injuries. These studies highlight the promising potential of PAI to evaluate and assess vascular changes caused by radiation.

Taken together, the existing proven capabilities of clinical PAI for the characterization of microvascular abnormalities and inflammatory reactions (136), suggests the potential for clinical application of the technology to assessment of radiation skin toxicity.

## Outlook

Preclinical studies have already indicated the promise of multi-scale PAI in radiation oncology, which motivates further research in both the preclinical and clinical settings. Accounting for the limited penetration depth of PAI, while ensuring clinical relevance, will require targeting accessible sites such as H&N, breast or skin lesions and associated superficial lymph node masses. In the preclinical setting, validation of PAI biomarkers in clinically relevant EBRT schedules for specific human cancer models is needed. Since the timings of intratumoral oxygenation modulation during EBRT fractionation show distinct profiles for different tumor models and fractionation schemes, longitudinal PAI assessment of response is needed to further guide clinical study designs and to assess the potential of both OE- and DCE-PAI biomarkers longitudinally.

In the clinical setting, to introduce PAI in the clinical simulation process of the radiotherapy workflow, end-to-end frameworks for in-room probe tracking with optimal co-registration software of PAI to planning CT need to be developed and validated. Furthermore, extensive assessment of biomarker reproducibility and repeatability will be needed before deployment for radiotherapy dose planning. Since photoacoustic signals are highly dependent on tissue properties in the light path of the imaged region of interest, accurate characterization of tissue absorption and light fluence effects at depth must be conducted if quantitative imaging biomarkers are to be derived. For instance, the impact of skin tone, or melanin concentration, on sO_2_ measurements needs to be assessed, since it can lead to image artefacts and incorrect estimations of chromophore concentrations in deeper tissues. Spectral coloring induced by the characteristic absorption of melanin in skin layers has been shown to impact the quantification of PAI biomarkers at depth in tissue *in silico* and in phantoms (137). Interestingly, a significant difference was reported in sO_2_ measured *in silico* for the same imaged object between the lightest and darkest tested pigmentation at the surface (137), with the same order of magnitude of the difference in arterial oxygenation saturation reported between white and black patients in a recent report assessing pulse oximetry biases to skin pigmentation (138). In a single wavelength system, the increased melanin concentration in the forearms of living subjects was associated with a decrease of PAI signal at depth and in a significantly different characterization of vascular structures in the skin (139). Understanding these effects *in vivo* is important and could enable a quantitative framework for data correction to be realized before PAI biomarkers become widely employed clinically.

Similarly, measurements taken with hand-held imaging modalities, such as ultrasound, are known to be operator-dependent. Both volume displacement and blood flow changes can be observed based on operator pressure in Doppler ultrasound and for different applications (140, 141). Such variability could be minimized in PAI through procedure standardization or by using non-handheld systems such as photoacoustic mammoscopes for breast imaging (101, 142), or a fixed probe on a mount with in-room infrared camera tracking, similar to previously developed and commercialized intrafraction ultrasound guidance systems (117). Variability induced by physiological processes such as breathing can also be controlled through breath-hold techniques or by tracking respiratory motion with optical surface guidance for instance (143), and the impact on imaging can be accounted for through intra-PAI co-registration with tomographic breathing detection (144). Trained radiographers and radiation therapists would have a key role in conducting these measurements and appropriate training would be essential in ensuring reproducibility. Preliminary assessment of PAI repeatability and reproducibility has been undertaken, suggesting good stability of repeated macroscopic PAI measurements *in vivo* (145). Future developments and cooperation between national and international bodies such as the International Photoacoustic Standardization Consortium (IPASC) (146, 147), the Quantitative Imaging Network (QIN) from the National Cancer Institute (148), and the Quantitative Imaging Biomarkers Alliance (QIBA) from the Radiological Society of North America (149) will be essential on the path of clinical translation (150).

Overall, PAI shows potential for providing predictive response biomarkers pre-EBRT and enabling assessment of vascular changes both in the tumor and in healthy irradiated skin after radiation exposure, highly relevant for detecting treatment response and modulating fractionated therapy. Furthermore, thanks to the non-ionizing nature and portability of PAI, these examinations could be repeated throughout treatment at bedside to enable longitudinal assessment of hypoxia during EBRT, especially for hypofractionated regimens such as SBRT and SABR. Integrating multi-scale PAI in radiation oncology with existing imaging modalities, from treatment guidance to early tumor response assessment and radiation toxicity, could therefore open new paradigms in the future of radiation oncology.

## Data Availability Statement

The original contributions presented in the study are included in the article/supplementary material. Further inquiries can be directed to the corresponding author.

## Author Contributions

All authors listed have made a substantial, direct, and intellectual contribution to the work and approved it for publication.

## Funding

All authors were supported by the Cancer Research UK under grant numbers C14303/A17197, C9545/A29580, C47594/A16267, C197/A16465, C47594/A29448 and in particular by the Cancer Research UK RadNet Cambridge under grant number C17918/A28870. TL is supported by the Cambridge Trust. LH is funded from NPL’s MedAccel programme financed by the Department of Business, Energy and Industrial Strategy’s Industrial Strategy Challenge Fund.

## Conflict of Interest

SB has previously received research funding from PreXion Corporation, which (Photoacoustic imaging division) was later acquired by CYBERDYNE Inc. and research support from iThera Medical GmbH, both vendors of photoacoustic imaging equipment. MT would like to disclose that he is currently employed at Merck & Co.

The remaining authors declare that the research was conducted in the absence of any commercial or financial relationships that could be construed as a potential conflict of interest.

## Publisher’s Note

All claims expressed in this article are solely those of the authors and do not necessarily represent those of their affiliated organizations, or those of the publisher, the editors and the reviewers. Any product that may be evaluated in this article, or claim that may be made by its manufacturer, is not guaranteed or endorsed by the publisher.
